# Relationship between daily pain and affect in women with rheumatoid arthritis: lower optimism as a vulnerability factor

**DOI:** 10.1007/s10865-017-9874-7

**Published:** 2017-07-17

**Authors:** Zuzanna Kwissa-Gajewska, Ewa Gruszczyńska

**Affiliations:** Health Psychology Department, SWPS University of Social Sciences and Humanities, Chodakowska 19/31, 03-815 Warsaw, Poland

**Keywords:** Rheumatoid arthritis, Optimism, Pain, Affect, Daily diary method

## Abstract

The aim of the study was to examine the moderating effect of optimism on the relationship between daily pain-daily affect. Fifty-four female patients with rheumatoid arthritis completed self-report measures of optimism (once), daily pain and daily positive and negative affect for 7 consecutive days during hospitalization. Results of multilevel random coefficients modeling demonstrated a significant cross-level interaction for daily negative affect only. Simple slopes analysis revealed that low optimism was related to a stronger positive relationship between daily pain and daily negative affect, whereas this effect was insignificant for higher optimism. High optimism was also related to higher daily positive affect, regardless of pain level. These findings suggest that low optimism may be a vulnerability factor in the daily pain-daily affect relationship rather than high optimism acting as a protective factor.

## Introduction

Rheumatoid arthritis (RA) is an autoimmune disease characterized by a progressive course of inflammation and stiffness of the joints, resulting in increasing functional disability (Taylor, 2012, p. 372). From a clinical point of view, it is a heterogeneous disease with hardly predictable outcomes, with a possible relapsing–remitting course (Eberhardt & Fex, 1998). This is why it may result in both pain-related immediate negative affect (Hamilton et al., 2005) as well as long-lasting negative mental health-related consequences, such as anxiety (Pincus et al., 1996) and depression (Dickens et al., 2002). Pain is reported by patients as a crucial and distressing symptom of RA (Jia & Jackson, 2016). According to a biopsychosocial model, pain is a result of a subjective evaluation of the nociception process that is modified not only by an individual biological composition but also by psychological and sociocultural factors (Gatchel et al., 2007). It is worth noting that most studies have failed to show a strong relationship between pain in RA and objective measures of inflammation (Egsmose & Madsen, 2015). Thus, individual differences related to emotional and cognitive processing may be important to the personal experience of pain.

Optimism has been defined as generalized positive outcome expectancies (Scheier & Carver, 1985); thus, it may be regarded as a relatively stable disposition, which is especially important in the context of stress and coping (Carver et al., 2010). So far, research has shown the significance of optimism for psychological well-being (Carver et al., 2010; Gatchel et al., 2007; Pakenham & Rinaldis, 2001; Thomas et al., 2011; Treharne et al., 2007), physical health (Brenes et al., 2002; Carver et al., 2010), immunological correlates, and objective markers of health status (Cohen et al., 1999; Rasmussen et al., 2009), as well as for pain intensity (Geers et al., 2008). In general, it has been proposed (cf. Scheier & Carver, 1985) that dispositional optimism acts as a buffer against adverse effects during a period of stress, but this effect has not been clearly defined. It has yet to be determined whether optimism is related to an increase of positive health outcomes or to a decrease of negative health outcomes under high stress or to both at the same time. Some results have indicated that optimism weakens the stress–negative outcomes link (Lai, 2009; Segerstrom, 2006), while others have indicated that that it strengthens the stress–positive outcomes link (Denovan & Macaskill, 2016). However, few examples exist of both kinds of effects being examined in one study (Atienza et al., 2002; Chang, 1998). In addition, it should be noted here that many studies on the moderating role of optimism have revealed only one main effect of optimism on health: that it is beneficial regardless of stress level (Treharne et al., 2007).

When it comes to the relationship between pain and affect, this buffering effect may be expressed as follows: the higher the optimism, the less positive the relationship between pain and negative affect, and the less negative the relationship between pain and positive affect. However, the nature of these relationships raises two issues: first, the effects of optimism on negative and positive affect can differ in magnitude, and second, these differences may vary with the level of optimism.

In other words, the effect of optimism on health-related outcomes can be symmetrical or asymmetrical in two ways: noted only for one kind of outcome (negative or positive affect) or noted only for a certain level of optimism (low vs. high). Thus, the results of a significant moderation effect may not necessarily indicate that high optimism is a *protective* factor (enhancing positive affect and reducing negative affect), but rather the opposite, that low optimism is a *vulnerability* factor (reducing positive affect and enhancing negative affect).

The majority of the studies mentioned so far have suggested that this effect may indeed be rather asymmetrical, albeit dependent on the study context and method (Grote & Bledsoe, 2007; Lai, 2009; Pakenham & Rinaldis, 2001; Segerstrom, 2006; Thomas et al., 2011). The inconsistency of these results can be attributed to the limitations of the study designs. First, they included mostly one type of outcome, representing negative dimensions of well-being. Second, they did not consider the symmetry of the optimism effect as a research question. Third, they included diverse indicators of stress. Finally, most of the studies were based on cross-sectional or classical longitudinal designs, which evaluate changes during longer periods of time and therefore rely strongly on participants’ retrospection. When it comes to pain, this recall memory can be especially biased (Affleck et al., 2001).

So-called intensive longitudinal studies have been developed over the past few decades (Bolger et al., 2003) to overcome this limitation and improve the ecological validity of measurements. The design of this type of studies is based on different forms and repetitions of daily diary reports to examine day-to-day variability when studying people in their natural setting. Due to a series of repeated measures, these studies allow researchers to separate a between-person variance from a within-person variance, taking into account that people differ from each other, but also that the same person may react differently over time (Bolger & Laurenceau, 2013). When applied to pain, this means that people may have a relatively stable tendency to report a sudden level of pain, but even then, pain reported at one moment may be lower or higher than is typical for this person. Thus, this approach allows researchers to obtain a richer accounting of the person’s affective experiences (Zautra & Sturgeon, 2016).

The majority of research on pain within the daily diary framework has concentrated on the pain–affect relationship. However, much of this research has only analyzed negative affect; positive affect has rarely been examined in pain context. As may be expected, negative mood increases on more painful days among RA patients (Affleck et al., 1992; 1999). Also, morning pain was related to evening pain among patients with RA, and this relationship was moderated by morning negative mood (Newth & DeLongis, 2004). In a few studies in which positive affect has been taken into account, results have revealed that during low-stress weeks, pain was related only to an increase of negative affect, whereas during high-stress weeks, pain was related to both an increase of negative affect and a decrease of positive affect (Davis & Zautra, 2004). Thus, it can be argued that these two types of affect should be analyzed separately in the context of pain, although the relationship between them can be modified by the patient’s pain level. Including optimism in this picture enables researchers to examine whether this personality disposition modifies their affective response to daily pain over time, even after controlling for a typical pain level for a given person. Previous findings have identified only a main effect of high optimism on better daily mood among RA patients, not a moderating effect (Affleck et al., 2001; Tennen et al., 1992), but this analysis includes a positivity ratio (a ratio of positive to negative affect), rather than two different types of affects (Tennen et al., 1992). The unique effects of optimism on each affect valence under chronic pain conditions has not been yet tested in daily diary studies.

### The current study

Thus, the main aim of the present study is to add to the current knowledge through a systematic examination of whether the stress-buffering effect of optimism on the daily pain–daily affect relationship is present for both affect valences and how it is distributed within an optimism disposition. If optimism is a protective factor, it should *reduce negative affect* but also act as *a buffer against positive affect reduction* in the face of pain. Thus, during higher than typical levels of pain for a given person, both a lower increase in negative affect and a lower decrease in positive affect should be observed for patients with higher levels of optimism. When this effect is noted only for negative or positive affect, it can hardly be described as protective, since both affect valences are crucial for maintaining well-being during chronic illness. Prolonged low negative affect itself, when not supported by an accurate intensity of positive affect, may still be a risk factor for depression (Wichers et al., 2007), which is one of the most frequently noted psychopathological symptoms among RA patients (cf. Taylor, 2012; p. 373). Thus, if a given person is experiencing higher than typical daily pain and either a higher increase in negative affect or a higher decrease in positive affect is observed, but this is only observed in patients low in optimism (i.e. with no symmetric effects for patients high in optimism), it suggests that low optimism is a vulnerability factor.

Finally, situations of hospitalization can be analyzed within the scope of critical life events. Even if not life-threatening in the case of RA, hospitalizations can still be regarded as uncontrollable and strongly interfere with a person’s other social roles and plans (Taylor, 2012, p. 226), requiring an adaptation effort. As the potential to change the situation through direct actions is very limited, the role of dispositional personal resources, such as optimism, should be crucial in such context. The high lifetime hospitalization rate among RA patients (Michet et al., 2015) makes this problem particularly valid clinically.

To sum up, the following hypotheses were tested:

1. Compared with low-optimism individuals, high-optimism individuals would report a weaker positive relationship between daily pain and daily negative affect, after controlling for the typical pain level for a given person.
2. Compared with low-optimism individuals, high-optimism individuals would report a weaker negative relationship between daily pain and daily positive affect, after controlling for the typical pain level for a given person.

Only the positive verification of both hypotheses will support the buffering effect of optimism in the pain–affect relationship.

## Method

### Participants

The sample consisted of 54 female patients aged 24–65 years (M = 52.07; SD = 9.91). Only women were selected because RA affects women more often than men, with a sex ratio of 4:1 (Harrison, 2003). Seventy-six percent of participants were married or partnered, and most of them (66.7%) reported having at least a high school education. The mean time since physician-confirmed diagnosis of RA was 11.43 years (SD = 8.41). The mean duration of morning stiffness was approximately 97 min (M = 96.45; SD = 139.21). According to recent findings, morning stiffness could be regarded as a clinically significant proxy of disease control and functional disability (Lindqvist et al., 2002; van Nies et al., 2015). Also, it has been found to have a stronger impact on patients’ well-being and ability to work than disease activity, as assessed by swollen joints and markers of inflammation (Yazici et al., 2004). The most frequent comorbid illness was osteoarthritis (50%). All sociodemographic and clinical data are presented in Table 1.

**Table 1:** Sociodemographic and clinical characteristics of participants (N = 54)

Variable	N (%)
Age in years (M ± SD)	52.07 ± 9.91
Duration of RA (years)	11.43 ± 8.41
Morning stiffness (yes)	52 (96.3%)
Duration of morning stiffness (minutes)	96.45 ± 139.35
Presence of other diseases	30 (55.6%)
Osteoarthritis	27 (50%)
Lupus erythematosus	3 (5.6%)
Marital status	
Married/cohabited	41 (76%)
Single	13 (14%)
Children (yes)	49 (90.7%)
Education	
Elementary school education	4 (7.4%)
Basic vocational education	14 (25.9%)
High school education	23 (42.6%)
University education	13 (24.1%)
Material status^a^ (M ± SD)	1.83 ± 0.42

*M* mean, *SD* standard deviation

^a^Material status was assessed subjectively on a three-point rating scale (1—*below average,* 2—*average*, 3—*above average*)

### Procedure

The study was approved by the institutional bioethical committee. Inclusion criteria were as follows: female, 18–65 years of age, a physician-confirmed diagnosis of RA, no severe comorbid somatic conditions other than those that are directly RA-related, RA flare as a cause for current hospitalization, an ability to perform basic usual self-care activities, and (due to the study protocol) possession of a mobile telephone.

The recruitment process lasted for 6 months and took place at a medical university hospital specializing in the diagnosis and treatment of RA. Sixty-three patients were approached, of whom 54 (85.71%) agreed to participate in the study. After obtaining an informed consent, participants obtained an initial packet of questionnaires for the measurement of optimism and basic sociodemographic and clinical information, which were completed and returned that same day. Then, participants received coded envelopes with a packet of “paper and pencil” diary questionnaires to report pain and positive and negative affect for seven consecutive evenings during hospitalization. They were reminded to do so by a short text message each evening. Thus, it was a time-fixed design (Bolger et al., 2003). The filled diaries were collected by a research assistant the next day.

### Measures

#### Dispositional optimism

Optimism was assessed only once, at the baseline, with the Life Orientation Test-Revised (LOT-R) by Scheier et al. (1994). The LOT-R is a 10-item measure of generalized positive outcome expectancies, including six items measuring dispositional optimism and four filler items. Responses are rated on a 5-point Likert-type scale, ranging from 0 *(strongly disagree)* to 4 *(strongly agree).* The Cronbach’s alpha for the present study was 0.71.

#### Positive and negative affect

Daily affect was assessed each day with a 20-item version of the Positive and Negative Affect Schedule (PANAS) by Watson et al. (1988). Participants were asked to rate on a 5-point scale, ranging from 1 (*not at all or a little*) to 5 (*extremely*), how they felt during the day using the scale consisted of 10-item positive affect and 10-item negative affect subscales. The mean internal reliability for these subscales over seven assessments was 0.92 and 0.93 for negative and positive affect, respectively.

#### Pain

Daily pain intensity was rated each day on a single 10 cm numeric pain rating scale by selecting a number between 0 (*no pain*) and 10 (*pain as bad as it has ever been*) that “describes your level of pain today” (Katz & Melzack, 1999).

### Data analysis

A multilevel model for diary longitudinal data was employed using IBM SPSS 24. The data included two levels: a within-person level (i.e. level 1, at which variables were expected to vary within each person over time) and a between-person level (i.e. level 2, at which variables were expected to vary from person to person).

Optimism in the study was measured only as a level 2 variable and was centered on the grand mean to facilitate interpretation (Hayes, 2006). The same rescaling procedure was applied to level 2 covariates. For daily assessments of pain, more complex transformations were required to separate variance related to level 1 and level 2 of this variable. Following the STARTS framework, proposed by Kenny and Zautra (2001), the level-2 pain was calculated by aggregating individual pain scores over 7 days, which resulted in a stable value for each person. This value was then subtracted from the observed daily pain score, which resulted in residual pain values. Thus, two orthogonal components were obtained, with level 1 interpreted as daily changes from the mean pain typical for a given person (“state-like”) and level 2 interpreted as individual differences in pain between persons (“trait-like”). Additionally, as with all other level-2 predictors, level-2 pain was centered on the grand mean.

Time was included in the model to control for its possible effect on affect. It was coded 0 for the first day of data collection and then from 1 to 6 accordingly for the next days. Models for positive and negative affect were analyzed separately, as they are assumed to be separate affective dimensions instead of poles of the same dimension. To control for autodependency between variables assessed day by day, which is typical for an intensive longitudinal design, an autoregressive covariance structure (AR1) was implemented at level 1. For level 2, an unstructured covariance matrix was assumed to examine possible random effects for intercepts and slopes.

The effects of cross-level interaction (simple slopes as outcomes) were calculated following the approach proposed by Preacher et al. (2006), supported by an online calculator (Preacher et al., 2004).

## Results

### Missing data analysis and descriptive statistics

The analysis consisted of 54 patients × 7 days = 378 observations. Missing data analysis reveals that participants filled out 371 (98.15%) of 378 diaries. None of the participants provided less than three diaries. There were no systematic changes between those who completed all the diaries and those who had a lower rate of compliance in terms of sociodemographic, clinical, and psychological variables. Thus, the expectation maximization method of missing data imputation was implemented (Enders, 2010). Descriptive statistics for variables are found in Table 2. Aggregated mean values of positive affect are significantly higher than those of negative affect (*t* = 14.48, *df* = 377, *p* < 0.001). Their correlation was negative but weak (*r* = −0.19).

**Table 2:** Descriptive statistics

Variable	M	SD	Range
Negative affect	16.40	6.44	10–46
Positive affect	25.09	8.58	1–47
Pain	4.50	2.32	0–10
Optimism	14.33	4.55	0–23

Results for aggregated data of 54 patients observed for 7 days

*M* mean, *SD* standard deviation

### Hypothesis 1: optimism as a moderator of daily pain effect on daily negative affect

Table 3 presents the results for multilevel modeling of negative affect as a function of pain and optimism after controlling for disease duration, morning stiffness, presence of other diseases, and time. As shown in the upper panel of Table 3, which illustrates fixed effects, there are significant main effects of daily pain and between-person differences in pain level; as expected, higher than average values of both are related to higher negative affect.

**Table 3:** Parameter estimates for multilevel model of negative affect as a function of pain and optimism

Fixed effects	Estimate	SE	t	*p*	95% CI	
					Lower	Upper
Intercept	15.64	0.83	18.93	<.001	13.99	17.29
Time	−0.34	0.10	−3.52	0.001	−0.53	−0.15
Disease duration	−0.01	0.06	−0.12	0.909	−0.13	0.12
Morning stiffness duration	−0.01	0.00	−1.64	0.108	−0.01	0.00
Presence of other diseases	2.83	0.89	3.19	0.003	1.04	4.62
Optimism	−0.25	0.12	−2.03	0.051	−0.50	0.00
Between-person pain	1.18	0.26	4.54	<.001	0.66	1.70
Within-person pain	0.87	0.23	3.86	<.001	0.41	1.33
Optimism × within-person pain	−0.11	0.05	−2.29	0.028	−0.20	−0.01

Random effects	Estimate	SE	z	*p*	95% CI	
					Lower	Upper
Level 1 (within-person)						
Residual	11.66	1.2	10.46	<.001	9.67	14.07
Autocorrelation	0.09	0.09	1.11	0.268	−0.07	0.26
Level 2 (between-person)						
Intercept	13.45	3.18	4.23	<.001	8.64	21.37
Within-person pain	0.80	0.41	1.93	0.053	0.29	2.21
Intercept and within-person pain	2.19	0.90	2.44	0.014	0.44	3.95

N = 54 patients, 7 days, 378 observations

**Fig. 1:**
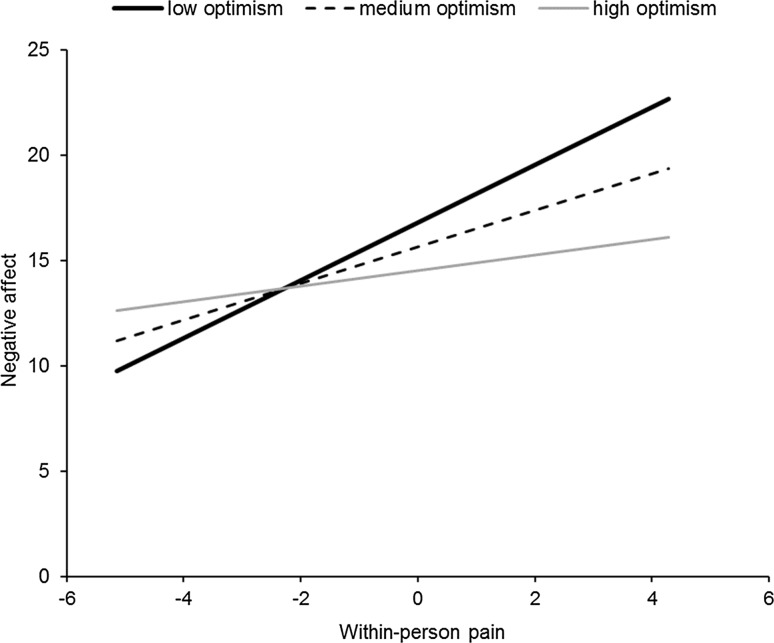
Simple regression slopes for optimism × within-person pain on negative affect. Within-person pain was centered around individual means, thus zero denotes the pain level typical for a given person during the time of the study. For optimism values described as mean, low and high indicates a sample average and 1 standard deviation below or above, respectively

Also, a significant time effect was noted, with negative affect decreasing over the time of the study. The single most important result here, however, is a significant cross-level interaction between daily pain and optimism. The simple effects of this interaction are plotted in Fig. 1. As optimism decreases, the slope of daily pain to negative affect becomes more strongly positive. The simple slope is 1.37 (*z* = 5.15, *p* < 0.001) at 1 SD below the mean of optimism, 0.87 (*z* = 3.78, *p* < 0.001) at the mean of optimism, and 0.37 and insignificant (*z* = 1.08, *p* = 0.3) at 1 SD above the mean.

**Fig. 2:**
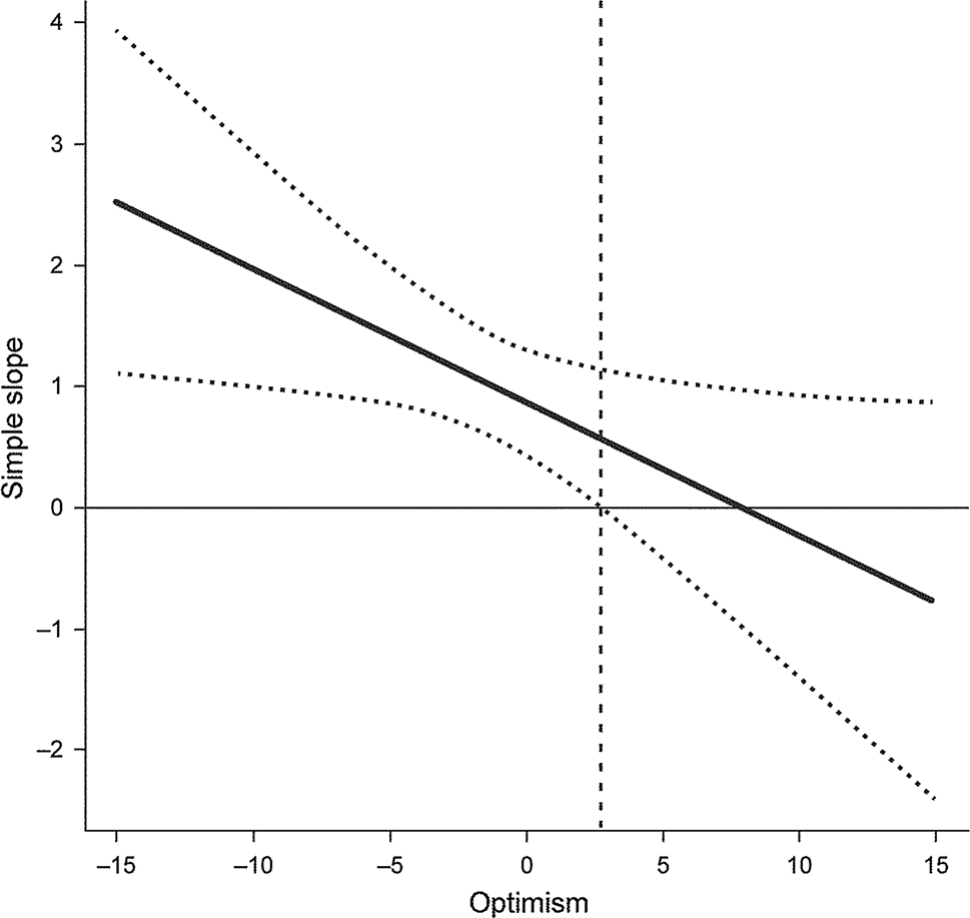
Confidence bands (*dotted lines*) for observed sample values of optimism with regard to within pain-negative affect slope. Optimism was centered around the sample mean, thus zero denotes mean value for the sample. The vertical *dashed line* indicates value of optimism at which confidence bans start to include simple slope of zero

Further inspection of confidence intervals, plotted in Fig. 2, reveals that the simple slope of daily pain regressed on negative affect is significantly different from zero for values of (centered) optimism above 2.78, which is approximately 2/3 of the SD (see Table 3) above the mean optimism value for the sample. This indicates that this relationship turns out to be insignificant at values of optimism only slightly above the mean.

Additionally, the lower panel of Table 3 indicates that significant between-person variability of negative affect intercepts can be observed as well as a significant relationship between intercept and daily pain slopes; the relationship between daily pain and negative affect is stronger among participants with a higher negative affect. In addition, there is a significant level-1 residual variance; however, no autocorrelation was observed between residuals.

### Hypothesis 2: optimism as a moderator of daily pain effect on daily positive affect

A similar analysis for positive affect revealed only significant main effects (upper panel of Table 4). Positive affect is higher on days with lower than typical pain for a given person and for those with higher than the sample average level of optimism. However, no significant interaction between these variables was shown. Also, substantial between-person variability in positive affect intercepts and daily pain slopes can be observed as well as significant level-1 residual variance and autocorrelations between residuals.

**Table 4:** Parameter estimates for multilevel model of positive affect as a function of pain and optimism

Fixed effects	Estimate	SE	t	*p*	95% CI	
					Lower	Upper
Intercept	24.68	1.39	17.73	<.001	21.89	27.46
Time	0.16	0.14	1.18	0.240	−0.11	0.43
Disease duration	−0.04	0.11	−0.40	0.694	−0.26	0.17
Morning stiffness duration	0.00	0.01	0.34	0.738	−0.01	0.01
Presence of other diseases	−0.16	1.53	−0.11	0.917	−3.25	2.93
Optimism	0.52	0.21	2.45	0.018	0.09	0.94
Between-person pain	−1.02	0.44	−2.31	0.026	−1.92	−0.13
Within-person pain	−1.08	0.30	−3.66	0.001	−1.68	−0.49
Optimism × within-person pain	0.11	0.06	1.79	0.080	−0.01	0.23

Random effects	Estimate	SE	z	*p*	95% CI	
					Lower	Upper
Level 1 (within-person)						
Residual	21.26	2.08	10.25	<.001	17.56	25.74
Autocorrelation	0.22	0.07	3.25	0.001	0.09	0.35
Level 2 (between-person)						
Intercept	39.75	9.08	4.38	<.001	25.41	52.19
Within-person pain	1.36	0.59	2.29	0.022	0.58	3.21
Intercept and within-person pain	−4.50	2.32	−1.94	0.053	−9.05	0.05

N = 54 patients, 7 days, 378 observations

Furthermore, the pattern of results remained unchanged after control for both main and interaction effects of individual differences in affect of an opposite valence to the model outcome variable.

## Discussion

Previous studies have demonstrated a buffering role of optimism for pain-related outcomes, but only in a few cross-sectional (Atienza et al. 2002; Chang, 1998; Lai, 2009) or longitudinal studies (Thomas et al., 2011). Thus, the aim of the presented study was to examine this effect with regard to the daily pain relationship with daily negative and positive affect yielding from 7 days of daily diaries provided by RA female patients during hospitalization. It was hypothesized that a positive relationship between daily pain and daily negative affect, as well as a negative relationship between daily pain and daily positive affect, would be weaker for patients with a higher level of optimism compared to those with a lower level of optimism. Thus, we expected a symmetrical effect of optimism for both negative and positive affect at the daily level. Those hypotheses were only partially supported. The significant optimism-daily pain interaction was revealed only for negative affect. Thus, the first major finding of study is that the effect of optimism on the daily pain–daily affect relationship may depend on affect valence.

In addition, the analysis of simple effects showed that individuals low in optimism reported greater same-day increase in negative affect in response to increase in pain intensity, but this effect turned out to be insignificant at values of optimism only slightly above the sample mean. This suggests another asymmetry: low optimism is a vulnerability factor rather than high optimism as a protective factor. This parallels earlier findings of cross-sectional and longitudinal studies in which a high level of experienced stress was positively related to negative health outcomes at low optimism values, but this effect became zero at a sudden point and *did not further decrease* with higher values of optimism (Grote & Bledsoe, 2007).

 Taken together, these two findings, i.e. a lack of moderating effect of optimism on the daily pain-positive affect relationship and a lack of negative affect reduction in response to daily pain with higher values of optimism, may undermine a buffering role of optimism in dealing with chronic pain. It is worth nothing that the average sample of pain intensity was moderate. Therefore, a lack of the aforementioned effects cannot be attributed to severe pain intensity and the high distress level this generally causes. Also, it is noteworthy that the female patients in our study reported higher average positive affect than negative affect, which was also observed in other studies with similar RA samples and design (Affleck et al., 1999; Sturgeon & Zautra, 2013). Additionally, the obtained effects were controlled for between-person differences in pain during the time of the study, and potentially pain-related covariates as well as random effects were included in the models to reflect the natural heterogeneity of the sample and to adjust for the interdependence of within-person data. Finally, since hospitalization can be regarded as a situation with highly similar objective characteristics for each participant, it could have been expected to facilitate the detection of a personality disposition effect on emotional reactions in response to pain (Wilson-Bernett & Carrigy, 1978).

In spite of these expectations, only the main effect of optimism was noted in the study and only for positive affect. The same results have been obtained in previous works (Affleck et al., 2001) and provided evidence that optimism acts as a source of well-being (Scheier et al., 2001). These results also support the notion of Affleck et al. (2001), that individuals with greater optimism have better chronic pain outcomes because they are generally less attentive to pain and better able to adjust to life with a pain condition. Thus, it seems that optimism may act as a generalized and long-term resiliency resource rather than a protective factor to lessen the impact of day-to-day fluctuations of chronic pain on corresponding mood changes. This hypothesis requires further research.

Nevertheless, the current study has several limitations that should be considered in interpretations of the results. The sample size can raise doubts about the sufficiency of statistical power, which in diary studies is positively related to an increasing number of participants rather than to an increasing number of measurement points per participant (Bolger & Laurenceau, 2013). Bearing this in mind, however, the ecological validity of this study can still be assumed as promising: at least it informs that probability to detect hypothesized effects may also be asymmetrical. A 7 day protocol was implemented because of the hospitalization context and duration as well as to minimize the practice and fatigue effect (Raudenbush & Xiao-Feng, 2001). Also, daily diary measures were obtained as end-of-day reports and therefore may be biased by systematic retrospection error (Hedges et al., 1985), but this is still less than that in longer measurement intervals, which are typical for longitudinal designs. Additionally, including in the study design a separate assessment of negative and positive affectivity as personal dispositions may have changed the results. For instance, it has been observed that the effect of optimism turned insignificant when controlled for affectivity (Benyamini & Roziner, 2008), but since such a control has still been rather exceptional than systematic, further studies are needed. As a matter of fact, our results also raise the long-existing doubts that findings on optimism can be biased by an overlap with negative affectivity (Smith et al., 1989).

In summary, our findings revealed an asymmetric effect of optimism on affective responses to daily pain among hospitalized women with RA, which suggests that within such a short-term time frame (a given day), low optimism acts as a vulnerability factor rather than high optimism acting as a protective factor. This indicates that patients with a low level of optimism may be especially prone to reacting with a higher negative affect on days with a higher than typical pain level. As it could influence both the patient’s pain-related behaviors (Newth & DeLongis, 2004) and treatment decisions (Rathbun et al., 2013), these findings are of special clinical importance.
